# A positive relationship between weight-adjusted waist index and non-alcoholic fatty liver disease: a study on US adolescents

**DOI:** 10.3389/fmed.2024.1424667

**Published:** 2025-01-07

**Authors:** Xiaoling Cui, Yize Huang, Luyang Kang, Lianxiu Han, Weijie Sun, Kexing Han, Yufeng Gao

**Affiliations:** ^1^Department of Infectious Diseases, The First Affiliated Hospital of Anhui Medical University, Hefei, China; ^2^Department of Infectious Diseases, Tongling People’s Hospital, Tongling, Anhui, China

**Keywords:** non-alcoholic fatty liver disease, liver steatosis, adolescents, weight-adjusted waist index, BMI, waist circumference

## Abstract

**Background:**

Non-alcoholic fatty liver disease (NAFLD) is the most prevalent chronic liver condition in children, underscoring the urgent need for non-invasive markers for early detection in this population.

**Methods:**

We utilized survey data from the National Health and Nutrition Examination Survey (NHANES) 2017–2020 regarding liver ultrasound transient elastography (LUTE) for the diagnosis of NAFLD (dependent variable), and used multiple logistic regression models to explore the association between weight-adjusted waist circumference index (WWI) and the prevalence of NAFLD in US adolescents. Smoothing curves and threshold effect analyses were used to assess the non-linear association between the independent variables and the dependent variable. Subgroup analysis was conducted to pinpoint particularly susceptible subgroups within our study cohort of 1,711 participants.

**Results:**

Our findings indicated a positive correlation between WWI and NAFLD scores. Adjusting for all covariates revealed a significant association between increased WWI and the presence of NAFLD, with an odds ratio of 3.37 (95% CI: 2.74, 4.15). This association proved stronger than those observed with waist circumference, body mass index, and NAFLD. Stratifying WWI into quartiles showed a clear and strong positive correlation (*P* for trend < 0.0001). The results of smoothing curves and threshold effect analysis showed a non-linear relationship between WWI and NAFLD (LLR < 0.001). Notably, for WWI values below 10.65, a significant correlation was observed (OR = 5.25, 95% CI: 3.77,7.31). Additionally, our subgroup analysis revealed that WWI and NAFLD were associated more positively among male participants aged 16 years and older.

**Conclusion:**

WWI is positively correlated with NAFLD in American adolescents and offers a straightforward and cost-effective method for identifying hepatic steatosis. The findings highlight the importance of focusing on individuals with a WWI below 10.65, where the risk of NAFLD increases. Priority should be given to the male adolescent population aged 16 and above.

## 1 Introduction

Non-alcoholic fatty liver disease (NAFLD) is characterized by fatty steatosis in more than 5% of liver cells, excluding alcohol consumption and other known causes (1, 2). It is now the most prevalent chronic liver disease in children and adolescents worldwide, with a prevalence rate of approximately 5%–11% (3, 4) and exceeding 50% in pediatric patients with other metabolic disorders (5). NAFLD can lead to progressive fibrosis and is a major contributor to the progression of end-stage liver disease, significantly increasing the demand for liver transplants (6, 7). Therefore, early detection of NAFLD in children and adolescents is crucial. Liver biopsy is considered the most accurate method for diagnosing pediatric NAFLD; however, due to its invasive nature and the potential for sampling errors, there is a significant need for non-invasive diagnostic techniques.

Several factors contribute to the development of NAFLD in young people, including weight gain, insulin resistance, central obesity, and other potential influences (8). In 2016, the World Health Organization (WHO) reported that over 340 million children and teenagers aged 5–19 years were classified as overweight or obese, highlighting the escalating global obesity crisis. The incidence of metabolism-related NAFLD in children and teenagers increased from 19.34 million in 1990 to 29.49 million in 2017, indicating a significant rise over the years (9). Recent data show that the prevalence of NAFLD linked to obesity among children and teenagers is 23.0% in Europe, 39.7% in South America, and 52.1% in Asia (10). Additionally, some studies have indicated that the growing obesity epidemic is strongly associated with an increased incidence and severity of NAFLD in children (11, 12). Consequently, the relationship between obesity indicators and NAFLD in children and adolescents is a major area of interest for researchers worldwide.

Risk factors that can influence NAFLD are often not singular, but previous studies have confirmed that it is usually associated with obesity (13). However, a significant proportion of patients with NAFLD do not appear to be obese in appearance, which makes screening for NAFLD challenging (14). The metrics that can be used to respond to a physical examination for obesity are diverse, and many of them have been shown to correlate with NAFLD (15, 16). The body mass index (BMI), a traditional measure of obesity, may fail to accurately identify a significant number of individuals with lean body mass and visceral obesity, raising questions about its accuracy (14, 17, 18). Waist circumference (WC), although an indicator of abdominal obesity, does not distinguish between visceral and subcutaneous adipose tissues and has been shown to correlate more strongly with subcutaneous fat (19). WC, for example, has been shown to be independently associated with increased cardiovascular risk, but its ability to predict visceral adipose tissue at the individual level remains limited (20). In response, Park et al. (21) introduced a novel obesity index in 2018 called the weight-adjusted waist index (WWI). This index, which is derived from both BMI and WC, offers a more precise measure of visceral obesity (21, 22). Research has confirmed that the accumulation of visceral fat is linked to a higher incidence of metabolic and endocrine disorders (23, 24). Oladimeji et al. (25) found that WWI was associated with blood pressure in a young population. Additionally, Park et al. (26) identified a positive linear correlation between WWI and type 2 diabetes mellitus. Elevated WWI has also been associated with an increased risk of cardiovascular death and overall mortality (27–29). However, to date, there have been no reports examining the relationship between WWI and NAFLD in adolescents.

## 2 Materials and methods

### 2.1 Data collection

National Health and Nutrition Examination Survey (NHANES) is administered by the CDC’ National Center for Health Statistics (NCHS) and consists of questionnaires, physical examinations, and laboratory tests for selected participants. The physical examination module of the NHANES 2017–2020 survey data contained data from liver ultrasound transient elastography (LUTE). The NCHS Ethics Review Board approved the NHANES survey protocol, and all participants provided written informed consent. The study was exempt from ethical review because the NHANES database is available to the public.

### 2.2 Study participants

**FIGURE 1:**
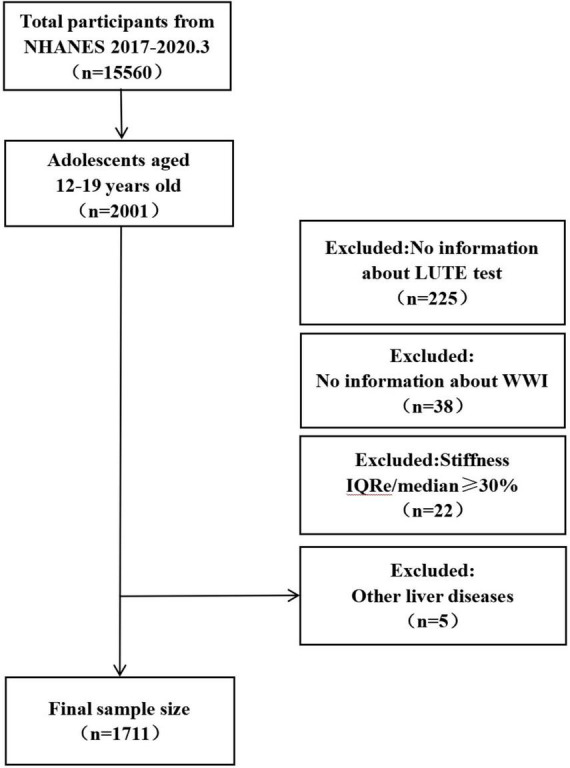
Flow chart for participants.

A total of 15,560 individuals were enrolled in the NHANES 2017–2020 cohort. In this cohort, participants aged 12 years and older were set to be able to take the FibroScan^®^ test. Based on the ages set for children and adolescents in previous study (6–11 years old for children; 12–15 years old for adolescents, and 16–19 years old for late adolescents) (30), the 12–19 year olds were selected for this study (*n* = 2001). Participants lacking complete information on the LUTE test were excluded (*n* = 225), as were those missing necessary laboratory parameters for calculating the WWI, including weight (*n* = 14) and WC (*n* = 24). Following NHANES guidelines, individuals with the ratio of interquartile range to the median stiffness (IQR/M) of 30% or higher for liver stiffness measurements were also excluded (*n* = 22). Additionally, participants diagnosed with any other liver conditions were excluded (*n* = 5). Ultimately, 1,711 individuals were enrolled in the study. Further details about the participants are presented in Figure 1.

### 2.3 Determination of NAFLD

Liver ultrasound transient elastography provides objective measurements of hepatic fibrosis (scarring in the liver) and hepatic steatosis (fat in the liver). Liver fibrosis is measured with the FibroScan^®^, which uses ultrasound and vibration-controlled transient elastography (VCTE™) to predict liver stiffness. The device also simultaneously measures the ultrasound attenuation associated with hepatic steatosis and records the Controlled Attenuation Parameter (CAP™) as an indicator of liver fat content (31). The ultrasound-based FibroScan^®^, which has been validated in multiple studies (32), is utilized for assessing liver steatosis. The collection of LUTE data in NHANES 2017–2020 required technicians to make at least 10 precise measurements, with the IQR/M of less than 30% as the primary data quality control criterion. The quality control criteria of IQR/M < 30% is to reduce variability and improve validity by taking measurements that result in few outliers. On this basis, and based on previous study, a median CAP of 248 dB/m or more is considered to be hepatic steatosis (4).

Importantly, during the period of our study (NHANES 2017–2020), the term NAFLD was used to describe the condition of fat accumulation in the liver without excessive alcohol consumption. Subsequent to our study period, in 2023, the terminology was updated to potentially include metabolic dysfunction-associated steatotic liver disease (MASLD), reflecting advancements in understanding the metabolic underpinnings of liver disease. However, as our analysis is based on NHANES data collected between 2017 and 2020, we have adhered to the NAFLD terminology prevalent during that time frame.

### 2.4 Calculation of WWI and BMI

Body mass index and WWI measurements were taken by certified medical professionals at the Mobile Exam Center (MEC). BMI is established by dividing the square of the height in meters by the weight in kilograms. In contrast, WWI is determined by dividing the square root of body weight (kg) by the WC (cm) (33). WC measurements were directly obtained from the recorded data. A review of NHANES body measurement data from 2017 to 2020 confirmed consistency across the study period.

### 2.5 Covariates

In previous studies, we adjusted the model to include additional variables (34, 35). Demographic variables include participant age, household income-to-poverty ratio (PIR) and race (classified as black, white, or other). Physical examination data encompasses height (cm), weight (kg), and WC (cm). Laboratory data comprises alanine aminotransferase (ALT) (U/L), gamma-glutamyl transaminase (GGT) (IU/L), uric acid (UA) (mg/dL), triglycerides (TG) (mg/dL), high-sensitivity C-reactive protein (HSCRP) (mg/L), white blood cell count (WBC) (1,000 cells/μl), platelet count (PLT) (1,000 cells/μl), ferritin (ng/ml), and glycohemoglobin (HbA1c) (%). To determine the presence of diabetes, prediabetes, and hypertension, participants were asked the following question: “Did your doctor tell you that you have diabetes, prediabetes, or high blood pressure?” In addition, we screened the selected covariates before constructing the final model. First, based on variance inflation factor (VIF), stepwise screening removed covariates with excessively high variances among them (VIF > 5) (Supplementary Table 1). Subsequently, according to the principle that the statistical significance *p*-value of regression coefficients introduced by the covariates should be <0.1 (Supplementary Table 2), the final included covariates were determined (Supplementary Table 3).

### 2.6 Statistical analysis

We utilized R and EmpowerStats to analyze all data in this study, and a *P*-value less than 0.05 was deemed statistically significant. The NHANES fieldwork was suspended in March 2020 due to the COVID-19 pandemic, resulting in an incomplete data collection cycle for 2019–2020. This disruption suggests that the findings may not fully represent the national population. To address this and create a nationally representative sample, data from the 2019 to March 2020 cycle was merged with information from the 2017 to 2018 NHANES cycle. We adhered to NHANES guidelines by using the check sample weights provided for the LUTE data analysis. Consequently, for the 2017–March 2020 period, special investigation sample weights (variable name: WTMECPRP) were used. The means and standard errors were utilized to depict continuous variables, while frequencies and percentages were employed to symbolize categorical variables. For continuous variables with no more than 10% missing values, missing data were imputed using the mean; otherwise, these variables were transformed into dichotomous variables using cutoffs defined by the reference ranges in the NHANES laboratory procedure manual, with missing values classified as a separate group. The association between NAFLD and independent variables (WWI, BMI, and WC) was assessed using multiple logistic regression models. Three models were adjusted: model 1 (unadjusted), model 2 (adjusted for age, race, and sex), and model 3 (adjusted for all covariates listed in Table 1). In order to identify sensitive individuals and evaluate the correlation between independent and dependent variables, subgroup analysis was then performed. In exploring the relationship between WWI and NAFLD, in order to ensure the accuracy and reliability of the results. We also paid special attention to the existence of non-linear relationships and their characteristics. First, we used smooth curve fitting analysis to confirm the existence of a non-linear relationship between WWI and NAFLD. This is because smoothed curve fitting analysis is a method of approximating data points by fitting a smooth curve, which could reveal underlying trends and patterns in the data, especially if the relationship between data points is not simply linear. Second, in order to determine the optimal inflection point (i.e., threshold point) among the independent variables of the WWI, we used threshold effects analysis. Threshold effect analysis is a method used to identify critical points where the relationship between variables changes significantly. Through this method, we were able to find the turning points when WWI affected NAFLD, which is critical to understanding how and when WWI began to significantly affect NAFLD. A log-likelihood ratio (LLR) of less than 0.05 was used to determine the significance of the non-linear relationships observed in the threshold effects analysis.

**TABLE 1:** Characteristics of participants.

Characteristic	Non-NAFLD group (*n* = 1,248)	NAFLD group (*n* = 463)	*P-*value
Gender (%)			0.074
Male	51.52	56.37	
Female	48.48	43.63	
Age (years)	15.30 ± 2.27	15.61 ± 2.17	0.011
Race (%)			0.007
White	54.33	61.99	
Black	26.68	19.87	
Other race	18.99	18.14	
PIR (%)			0.039
<1.35	35.74	39.74	
1.35–3.45	30.37	32.18	
>3.45	22.76	16.41	
Unclear	11.14	11.66	
Hypertension (%)			0.008
Yes	12.66	17.71	
No	87.34	82.29	
Diabetes and prediabetes (%)			<0.001
Yes	2.00	7.56	
No	97.92	91.79	
Unclear	0.08	0.65	
ALT (U/L) (%)			<0.001
Male ≥ 26, Female > 22	5.93	27.65	
Male < 26, Female ≤ 22	80.69	60.26	
Unclear	13.38	12.10	
GGT (U/L) (%)			<0.001
≤33	84.62	79.05	
>33	2.00	8.86	
Unclear	13.38	12.10	
UA (mg/dl) (%)			<0.001
≤7.0	82.77	74.08	
>7.0	3.85	13.82	
Unclear	13.38	12.10	
TG (mg/dl) (%)			<0.001
≤150	80.53	68.25	
>150	6.09	19.65	
Unclear	13.38	12.10	
HSCRP (mg/L) (%)			<0.001
<5	81.33	69.98	
≥5	5.29	17.71	
Unclear	13.38	12.31	
WBC (1,000 cells/μl)	6.69 ± 1.86	7.78 ± 2.15	<0.001
PLT (1,000 cells/μl)	262.02 ± 54.89	280.78 ± 57.36	<0.001
Ferritin (ng/ml)	54.46 ± 40.99	68.14 ± 60.88	<0.001
HbA1c (%)	5.26 ± 0.38	5.35 ± 0.65	<0.001
BMI (kg/m^2^)	22.63 ± 4.68	30.68 ± 7.51	<0.001
Under weight and normal weight (%)	70.83	19.87	<0.001
Overweight and obese	29.17	80.13	<0.001
WC (cm)	77.66 ± 11.23	98.46 ± 17.34	<0.001
WWI	9.94 ± 0.70	10.69 ± 0.77	<0.001

Median and quartiles for continuous variables: *P*-value was calculated by weighted linear regression model. % for categorical variables: *P*-value as calculated by weighted Chi-square test.

## 3 Results

### 3.1 Baseline characteristics of participants

This study included a total of 1,711 individuals, with an analysis of their general characteristics presented in Table 1. Statistically significant differences were noted in age, race, and PIR (*P* < 0.05) between the groups. Participants in the NAFLD group exhibited higher rates of diabetes, prediabetes, and hypertension. Furthermore, the NAFLD group had higher levels of ALT, GGT, UA, TG, HSCRP, WBC, PLT, ferritin, and HbA1c (*P* < 0.01), and a notable statistical disparity was observed in BMI, WC, and WWI between the two groups (*P* < 0.001).

### 3.2 The correlation between WWI and NAFLD

A positive correlation was observed between NAFLD and the variables BMI, WC, and WWI across all three models (models 1–3). The association between WWI and NAFLD was stronger than that between BMI and NAFLD, as well as WC and NAFLD. The final adjusted model showed that each unit increase in WWI increased the risk of developing NAFLD by 3.37% (OR = 3.37, 95% CI: 2.74, 4.15). This relationship persisted when WWI was categorized into quartiles, with the correlation strength increasing as WWI increased (*P* < 0.0001). In the highest quartile of WWI (10.57–13.27), a significant association was observed (OR = 11.65, 95% CI: 7.28, 18.65). Similarly, in the fourth quartile groups for BMI and WC, positive correlations were found between WC and NAFLD (OR = 20.69, 95% CI: 12.75, 33.59) and BMI and NAFLD (OR = 21.86, 95% CI: 13.26, 36.05). These results are summarized in Table 2.

**TABLE 2:** Association of BMI, WC, and WWI with NAFLD.

Exposure	Model 1 OR (95% CI)	Model 2 OR (95% CI)	Model 3 OR (95% CI)
**NAFLD**			
BMI (kg/m^2^)	1.25 (1.22, 1.28)	1.28 (1.24, 1.31)	1.25 (1.21, 1.29)
**Quartiles of BMI**			
Q1 (13.2–20.1)	Reference	Reference	Reference
Q2 (20.2–23.1)	1.66 (0.99, 2.76)	1.79 (1.07, 3.01)	1.73 (1.02, 2.94)
Q3 (23.2–27.7)	5.25 (3.34, 8.24)	5.72 (3.60, 9.09)	4.86 (3.02, 7.81)
Q4 (27.8–59.4)	29.32 (18.81, 45.71)	36.87 (23.07, 58.90)	21.86 (13.26, 36.05)
*P* for trend	<0.0001	<0.0001	<0.0001
WC (cm)	1.11 (1.09, 1.12)	1.11 (1.10, 1.12)	1.10 (1.09, 1.12)
**Quartiles of WC**			
Q1 (53.5–71.2)	Reference	Reference	Reference
Q2 (71.3–79.4)	1.31 (0.79, 2.18)	1.31 (0.79, 2.20)	1.21 (0.72, 2.04)
Q3 (79.5–92.0)	4.22 (2.73, 6.54)	4.28 (2.73, 6.70)	3.77 (2.37, 5.98)
Q4 (92.1–157.9)	30.85 (20.09, 47.39)	32.13 (20.61, 50.09)	20.69 (12.75, 33.59)
*P* for trend	<0.0001	<0.0001	<0.0001
WWI	3.78 (3.20, 4.47)	4.94 (4.09, 5.96)	3.37 (2.74, 4.15)
**Quartiles of WWI**			
Q1 (7.89–9.55)	Reference	Reference	Reference
Q2 (9.56–10.07)	1.69 (1.09, 2.65)	2.19 (1.38, 3.47)	1.94 (1.22, 3.11)
Q3 (10.08–10.56)	5.12 (3.43, 7.65)	8.61 (5.55, 13.36)	5.82 (3.70, 9.16)
Q4 (10.57–13.27)	14.07 (9.48, 20.87)	25.00 (16.05, 38.92)	11.65 (7.28, 18.65)
*P* for trend	<0.0001	<0.0001	<0.0001

Model 1: no covariates were adjusted; model 2: age, gender, and race were adjusted; model 3: all covariates were adjusted.

### 3.3 Analysis by subgroups of the correlation between WWI and NAFLD

To verify the robustness of the positive correlation between WWI and NAFLD in specific demographic groups and to identify particularly vulnerable cohorts, we conducted a subgroup analysis. In the fully adjusted model, the results demonstrated a positive correlation between WWI and NAFLD among male participants (OR = 4.61, 95% CI: 3.39, 6.26) and those aged 16 years and older (OR = 3.51, 95% CI: 2.56, 4.82). These findings are detailed in Table 3. When analyzing BMI as the dependent variable, a stronger positive correlation was observed between BMI and NAFLD in males (OR = 1.32, 95% CI: 1.26, 1.39) and in individuals aged 16 and above (OR = 1.29, 95% CI: 1.23, 1.35), as shown in Table 4. Similarly, the correlation between WC and NAFLD was consistent when WC was analyzed as the response variable, detailed in Table 5.

**TABLE 3:** Subgroup analysis of association between WWI and NAFLD.

Characteristic	Model 1 OR (95% CI)	Model 2 OR (95% CI)	Model 3 OR (95% CI)
**Stratified by gender**			
Male	4.59 (3.63, 5.80)	6.38 (4.84, 8.41)	4.61 (3.39, 6.26)
Female	3.86 (2.97, 5.01)	3.95 (3.00, 5.20)	2.68 (1.96, 3.65)
**Stratified by age (years)**			
<16	3.66 (2.90, 4.62)	3.83 (3.00, 4.88)	2.86 (2.19, 3.75)
≥16	4.14 (3.24, 5.30)	5.47 (4.14, 7.24)	3.51 (2.56, 4.82)
**Stratified by race**			
White	3.89 (3.10, 4.87)	5.22 (4.03, 6.75)	3.77 (2.83, 5.02)
Black	3.25 (2.39, 4.42)	4.23 (2.94, 6.09)	3.08 (1.99, 4.77)
Other race	4.44 (2.87, 6.85)	6.23 (2.66, 9.79)	3.98 (2.26, 7.02)

Model 1: no covariates were adjusted; Model 2: age, gender, race were adjusted. Model 3: all covariates were adjusted.

**TABLE 4:** Subgroup analysis of association between BMI and NAFLD.

Characteristics	Model 1 OR (95% CI)	Model 2 OR (95% CI)	Model 3 OR (95% CI)
**Stratified by gender**			
Male	1.34 (1.28, 1.40)	1.35 (1.29, 1.41)	1.32 (1.26, 1.39)
Female	1.21 (1.17, 1.24)	1.23 (1.19, 1.27)	1.21 (1.16, 1.26)
**Stratified by age (years)**			
<16	1.25 (1.20, 1.29)	1.26 (1.22, 1.31)	1.24 (1.19, 1.29)
≥16	1.26 (1.22, 1.31)	1.29 (1.24, 1.34)	1.29 (1.23, 1.35)
**Stratified by race**			
White	1.30 (1.25, 1.34)	1.31 (1.26, 1.36)	1.29 (1.24, 1.35)
Black	1.18 (1.13, 1.22)	1.20 (1.15, 1.25)	1.19 (1.13, 1.25)
Other race	1.37 (1.27, 1.48)	1.39 (1.29, 1.51)	1.36 (1.24, 1.49)

**TABLE 5:** Subgroup analysis of association between WC and NAFLD.

Characteristics	Model 1 OR (95% CI)	Model 2 OR (95% CI)	Model 3 OR (95% CI)
**Stratified by gender**			
Male	1.12 (1.10, 1.14)	1.12 (1.11, 1.14)	1.12 (1.10, 1.14)
Female	1.09 (1.08, 1.11)	1.10 (1.08, 1.12)	1.10 (1.07, 1.12)
**Stratified by age (years)**			
<16	1.10 (1.09, 1.12)	1.10 (1.09, 1.12)	1.10 (1.08, 1.12)
≥16	1.11 (1.10, 1.13)	1.12 (1.10, 1.13)	1.12 (1.09, 1.14)
**Stratified by race**			
White	1.12 (1.10, 1.13)	1.12 (1.10, 1.14)	1.12 (1.10, 1.14)
Black	1.07 (1.06, 1.09)	1.08 (1.06, 1.10)	1.08 (1.05, 1.10)
Other race	1.14 (1.11, 1.18)	1.15 (1.11, 1.19)	1.16 (1.11, 1.21)

### 3.4 Analysis of the threshold effect and smooth curve fitting for WWI with NAFLD

**FIGURE 2:**
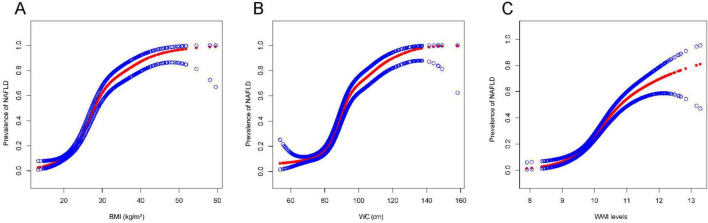
The association of BMI, WC, and WWI with NAFLD. Red band represents a smooth curve fit between the variables. The blue bands represent the 95% confidence intervals of the fitted results. The independent variables were BMI (kg/m^2^) **(A)**, WC (cm) **(B)**, and WWI **(C)**. All the covariates in Table 1 were adjusted.

We explored the possibility of a non-linear relationship between WWI and NAFLD using smooth curve fitting and threshold effect analysis. The analysis revealed that the relationship was not linear, as depicted in Figure 2. Using the threshold effect model, a non-linear link was identified between WWI and NAFLD, with an LLR less than 0.001. Notably, the positive association between WWI and NAFLD intensified while WWI was below 10.65, as indicated by the effect value (OR = 5.25, 95% CI: 3.77, 7.31), shown in Figure 2A. Conversely, when WC was the dependent variable, a slight increase in NAFLD risk was noted (OR = 1.11, 95% CI: 1.09, 1.13) when WC exceeded 73.3 (LLR = 0.046), as seen in Figure 2B. Additionally, a modest rise in the prevalence of NAFLD was observed (OR = 1.31, 95% CI: 1.26, 1.36) when BMI was below 31.4 and used as the dependent variable (LLR < 0.001), presented in Figure 2C. The outcomes of the smooth curve fitting and threshold effect analyses are also summarized in Table 6.

**TABLE 6:** Threshold effect analysis of the association of BMI, WC, and WWI with NAFLD.

Exposure	BMI	WC	WWI
**Linear model**			
OR (95% CI)	1.25 (1.21, 1.29)	1.10 (1.09, 1.12)	3.37 (2.74, 4.15)
**Non-linear model**			
Inflection point (K)	31.4	73.3	10.65
OR (95% CI) (<K)	1.31 (1.26, 1.36)	1.03 (0.96, 1.10)	5.25 (3.77, 7.31)
OR (95% CI) (>K)	1.12 (1.05, 1.19)	1.11 (1.09, 1.13)	1.73 (1.15, 2.61)
LLR	<0.001	0.046	<0.001

## 4 Discussion

Non-alcoholic fatty liver disease is a multisystemic disease that increases the risk of liver-related complications as well as morbidity and mortality associated with cardiovascular disease. In adults, obesity, particularly abdominal obesity, is a recognized risk factor for NAFLD. In our study population aged 12–19 years, 80.13% of adolescents were found to be overweight or obese (Table 1). This is consistent with the findings of Ruiz et al. (36), who noted that obesity disproportionately affects adolescents within this age group. BMI and WC are commonly used indicators to assess obesity, and numerous studies have shown that high BMI and WC are positively associated with the development of NAFLD (37–40). However, there are currently no reports on the association between WWI findings and NAFLD in adolescents.

Our study confirmed that WWI is more relevant than BMI and WC for assessing the occurrence of NAFLD in adolescents. Moreover, when WWI was categorized into quartiles, the positive correlation with NAFLD increased as WWI increased. NAFLD in adolescents is a metabolic stress-related liver injury strongly associated with obesity and insulin resistance (41). The pathogenesis of NAFLD involves the “multiple-hit doctrine,” which includes hepatic triglyceride deposition, inflammatory cytokines, free fatty acids, intestinal microecology, and genetic inheritance (42). Obesity is the most common cause of childhood and adolescent liver disease. Research has substantiated the strong correlation between obesity, metabolic disorders, and inflammation (43–45). Excess body fat, particularly visceral fat, promotes elevated blood pressure, insulin resistance, and atherosclerosis through the action of adipocytokines produced by the adipose tissue (46). Furthermore, adipocytokines enhance insulin resistance and induce systemic inflammation and oxidative stress, which promote the formation of NAFLD (47). Therefore, it appears that the initiation of obesity plays a critical role in the etiology of NAFLD and potentially in the progression to NASH.

Body mass index and WC are two common indicators of obesity (48). However, changes in the height and weight components of BMI may represent physiological rather than pathological states in children and adolescents. WC, while highly correlated with BMI, does not accurately capture the full extent of obesity. The standardized WWI has proven more accurate in assessing obesity and is more closely associated with morbidity and mortality from obesity-related diseases (28, 49). Hu et al. (37) reported a positive association between WWI and NAFLD in adults aged 20 years and older (37). Shen et al. (50) demonstrated an increase in the CAP of 7.6 dB/m for each unit increase in WWI among US adults. However, an association between WWI and NAFLD has not yet been reported in pediatric and adolescent populations. Our study showed that WWI was more accurate in predicting NAFLD in children and adolescents (OR = 3.37, 95% CI: 2.74, 4.15). Particularly, the correlation between WWI and NAFLD was more pronounced when WWI was below 10.65 (OR = 5.25, 95% CI: 3.77, 7.31).

From the subgroup analysis, we concluded that male adolescents aged 16 and older require particular attention in NAFLD screening. Multiple meta-analyses and epidemiological studies have found that the incidence of NAFLD is significantly higher in males than in females. A recent prospective cohort study showed a stronger positive correlation between abdominal adipose tissue and the cumulative incidence of NAFLD in men compared to women (51). Men have a lower risk of subcutaneous fat distribution in the buttocks and femurs and a higher risk of visceral obesity, which is mainly characterized in women by accumulation in the buttocks, limbs, and subcutaneous fat. Compared to abdominal adipose tissue, the lipolytic response of gluteal femoral fat to epinephrine and norepinephrine is lower, releasing fewer fatty acids, while estradiol reduces lipolysis, resulting in fewer fatty acids delivered to the liver. Body and visceral fat content increase with age. A cross-sectional study assessing NAFLD in 33,216 Spaniards aged 18–65 years showed that NAFLD prevalence was more pronounced in males and increased with age (52). Similar results were observed in a prospective cohort study of children and adolescents, where the prevalence of fatty liver was 2.38% in students under 8 years and 24.76% in students over 17 years, with a higher prevalence of severe fatty liver among high school students (53). Previous study demonstrated a correlation between low serum testosterone and NAFLD or even cirrhosis due to NAFLD and was associated with poor clinical outcomes (54), a feature that was more pronounced in male patients. In a cross-sectional study, WWI was shown to correlate with a decrease in total serum testosterone and was more pronounced in males in late adolescence (16–19 years old) (55). Therefore, we suggested that male adolescents aged 16 years and older may have attenuated the protective effect of testosterone against hepatic steatosis due to the reduction in total testosterone resulting from higher WWI.

To our knowledge, this is the first cross-sectional study to explore the association between WWI and NAFLD in pediatric and adolescent populations. According to the NASPGHAN clinical practice guidelines for the diagnosis and treatment of NAFLD in children (56), there are limitations with using ALT as the preferred first-line screening indicator due to its inadequate sensitivity and specificity (57, 58). Consequently, conventional ultrasound, which also lacks adequate sensitivity and specificity, is not recommended for NAFLD screening programs in children. Therefore, WWI has the potential to be used as a screening indicator for NAFLD in children and adolescents. However, there were some limitations to this study. First, the use of ultrasound fiber scanning for the diagnosis of NAFLD has some limitations. Although fiber scanning had been validated in several studies and was considered a reliable tool for assessing hepatic steatosis and fibrosis (35), it has its own limitations. For example, the CAP derived from fiber scans can estimate liver fat content but is not as accurate as liver biopsy, which remains the gold standard for diagnosing NAFLD. In addition, LUTE may be affected by factors such as liver inflammation, bile duct obstruction, and iron overload during operation, which may lead to misclassification in some cases (59). Meanwhile, inter- and intra-observer variability in CAP measurements, although generally low, cannot be completely ignored. All of these factors may lead to bias in our results, and thus further validation using more explicit diagnostic methods is needed in future studies. Second, the cross-sectional design of our study limited our ability to determine causality. Although our findings indicated a strong positive association between WWI and NAFLD, this design did not allow us to infer a causal relationship between the two. The currently observed association may be due to unmeasured confounders or reverse causality. For example, patients with NAFLD may have a change in body composition that leads to an increase in WWI, rather than the other way around. To address this limitation, longitudinal studies are necessary to track changes in WWI over time and its relationship to the onset and progression of NAFLD. Third, the generalizability of our findings may be limited by the specificity of the study population. Our study focused on adolescents aged 12–19 years in the United States, and the findings may not be applicable to other age groups or populations of different ethnicities and races. Future studies involving more diverse population groups are necessary to confirm the broad applicability of our findings. Finally, the COVID-19 pandemic disrupted data collection in 2020 and may have affected the representativeness of our sample. In conclusion, while our study provided valuable insights into the relationship between WWI and NAFLD in adolescents, further research is needed to address these limitations and confirm our findings. Prospective studies in larger, more diverse populations, combined with more definitive diagnostic methods, will help solidify the role of WWI as a potential screening tool for NAFLD in pediatric and adolescent populations.

## 5 Conclusion

This study found a non-linear positive association between WWI and NAFLD in US adolescents. This non-linear positive association was more significant at WWI below 10.65. The positive association between WWI and NAFLD was stronger in male adolescents aged 16 years and older. Thus, WWI is useful as a simple and economical indicator to identify hepatic steatosis in adolescents.

## Data Availability

The raw data supporting the conclusions of this article will be made available by the authors, without undue reservation.
