# Supplementary material for: A positive relationship between weight-adjusted waist index and non-alcoholic fatty liver disease: a study on US adolescents
Source: Front Med (Lausanne). 2025 Jan 7;11:1424667. doi: 10.3389/fmed.2024.1424667 (PMC11753237; doi:10.3389/fmed.2024.1424667)
Supplement: Supplementary file 1 [file Data_Sheet_1.doc]

Supplementary Table 1. Diagnosis of co-linearity of variables

| **Variables** | **Step 1** | **Step 2** | **Step 3** | **Step 4** | **Step 5** | **Step 6** | **Step 7** | **Step 8** | **Step 9** |
| --- | --- | --- | --- | --- | --- | --- | --- | --- | --- |
|  | VIF 1 | VIF 2 | VIF 3 | VIF 4 | VIF 5 | VIF 6 | VIF 7 | VIF 8 | VIF 9 |
| BMI(kg/m^2^) | 14.3 | 14.3 | 14.3 | 14.3 | 14.3 | 14.3 | 14.2 | 14.2 | 14.2 |
| WC(cm) | 20 | 20 | 20 | 20 | 20 | 20 | 19.9 | 19.9 | 19.9 |
| WWI | 3.3 | 3.3 | 3.3 | 3.3 | 3.3 | 3.3 | 3.3 | 3.3 | 3.3 |
| Gender | 2.2 | 2.2 | 2.2 | 2.2 | 2.2 | 2.2 | 2.2 | 2.2 | 2.2 |
| Age (yrs) | 3 | 3 | 2.9 | 2.9 | 2.9 | 2.9 | 2.9 | 2.9 | 2.9 |
| Race | 1.1 | 1.1 | 1.1 | 1.1 | 1.1 | 1.1 | 1.1 | 1.1 | 1.1 |
| PIR(%) | 1 | 1 | 1 | 1 | 1 | 1 | 1 | 1 | 1 |
| Diabetes or Prediabetes | 1.1 | 1.1 | 1.1 | 1.1 | 1.1 | 1.1 | 1.1 | 1.1 | 1.1 |
| Hypertension | 1.1 | 1.1 | 1.1 | 1.1 | 1.1 | 1.1 | 1.1 | 1.1 | 1.1 |
| ALT(U/L) | 3.3 | 3.3 | 3.3 | 3.2 | 3.1 | 3.1 | 2.9 | 2.9 | 2.7 |
| ALB (g/dl) | 2.7 | 2.7 | 2.7 | 2.7 | 2.7 | 2.6 | 2.6 | 2.6 | 2.5 |
| ALP (IU/L) | 4.5 | 4.5 | 4.5 | 4.5 | 4.4 | 4.4 | 4.3 | 4.3 | 4.3 |
| AST (U/L) | 6.2 | 6.2 | 6 | 5.9 | 5.9 | 5.5 | 5.3 | 5.2 | NA |
| Glucose (mg/dl) | 9.1 | 9.1 | 8.9 | 8.7 | NA | NA | NA | NA | NA |
| GGT (U/L) | 13.7 | 13.7 | NA | NA | NA | NA | NA | NA | NA |
| Serum iron (ug/dL) | 8 | 8 | 8 | 7.5 | 7.3 | 7.1 | NA | NA | NA |
| LDH（IU/L） | 4.7 | 4.7 | 4.7 | 4.7 | 4.6 | 4.6 | 4.6 | 4.6 | 3.9 |
| TBIL (mg/dl) | 10.8 | 10.8 | 10.3 | NA | NA | NA | NA | NA | NA |
| UA (mg/dl) | 8.9 | 8.9 | 8.5 | 8.2 | 7.9 | NA | NA | NA | NA |
| HbA1c (%) | 1.2 | 1.2 | 1.2 | 1.2 | 1.2 | 1.2 | 1.1 | 1.1 | 1.1 |
| WBC(1000 cells/UL) | 917 | NA | NA | NA | NA | NA | NA | NA | NA |
| LYM (1000 cells/UL) | 98.1 | 1.3 | 1.3 | 1.3 | 1.3 | 1.3 | 1.3 | 1.3 | 1.3 |
| MON(1000 cells/UL) | 8.3 | 1.7 | 1.7 | 1.7 | 1.7 | 1.7 | 1.7 | 1.7 | 1.7 |
| NEU (1000 cells/UL) | 584.9 | 1.7 | 1.7 | 1.7 | 1.7 | 1.7 | 1.7 | 1.7 | 1.7 |
| EOS(1000 cells/UL) | 6.7 | 1.1 | 1.1 | 1.1 | 1.1 | 1.1 | 1.1 | 1.1 | 1.1 |
| HGB（g/dl） | 2 | 2 | 2 | 2 | 2 | 2 | 2 | 2 | 2 |
| PLT (1000 cells/UL) | 1.3 | 1.3 | 1.3 | 1.3 | 1.3 | 1.3 | 1.3 | 1.3 | 1.3 |
| Ferritin (ng/ml) | 1.5 | 1.5 | 1.5 | 1.5 | 1.5 | 1.5 | 1.5 | 1.5 | 1.5 |
| TC (mg/dl) | 6.2 | 6.2 | 6.2 | 6.1 | 6.1 | 6 | 6 | NA | NA |
| HsCRP(mg/L) | 6 | 6 | 5.8 | 5.7 | 5.5 | 5.4 | 5.2 | 4.9 | 4.7 |
| TG (mg/dl) | 6.5 | 6.5 | 6.2 | 6.1 | 5.9 | 5.6 | 5.4 | 4.9 | 4.4 |
| HDL(mg/dl) | 2.5 | 2.5 | 2.5 | 2.5 | 2.5 | 2.5 | 2.5 | 2.2 | 2.2 |

*Excluded variables: AST (U/L)、Glucose (mg/dl)、GGT (U/L)、Serum iron (ug/dL)、TBIL (mg/dl)、UA (mg/dl)、WBC(1000 cells/UL)、TC (mg/dl)

Supplementary Table 2. Covariates Screening

| **Covariates** | **β** | **SE** | **95%CI Low** | **95%CI Upp** | **P value** |
| --- | --- | --- | --- | --- | --- |
| Gender | | | | | |
| Male | Reference | | | | |
| Female | -9.0861 | -0.4610 | 0.6637 | 1.0195 | 0.0744 |
| Age (yrs) | | | | | |
| 13 | 0.2371 | 0.5949 | 0.8010 | 2.0294 | 0.3056 |
| 14 | 0.2238 | 0.3982 | 1.1794 | 2.8355 | 0.0070 |
| 15 | 0.2320 | 0.6097 | 1.2280 | 3.0491 | 0.0044 |
| 16 | 0.2327 | 0.0906 | 0.9638 | 2.3996 | 0.0716 |
| 17 | 0.2295 | 1.2093 | 1.0968 | 2.6972 | 0.0181 |
| 18 | 0.2343 | 0.8177 | 1.2074 | 3.0249 | 0.0057 |
| 19 | 0.2371 | 0.7145 | 1.0881 | 2.7814 | 0.0207 |
| Race | | | | | |
| White | Reference | | | | |
| Black | 0.1372 | 0.9067 | 0.4987 | 0.8541 | 0.0019 |
| Other Race | 0.1452 | -0.0224 | 0.6299 | 1.1130 | 0.2213 |
| PIR | | | | | |
| <1.35 | Reference | | | | |
| 1.35-3.45 | -0.3365 | 0.2397 | 0.4465 | 1.1427 | 0.1605 |
| >3.45 | -1.0824 | 0.3535 | 0.1694 | 0.6774 | 0.0022 |
| Unclear | -0.2407 | 0.3331 | 0.4092 | 1.5103 | 0.4701 |
| Diabetes or Prediabetes | | | | | |
| Yes | Reference | | | | |
| No | -1.4762 | 0.3407 | 0.1172 | 0.4455 | <0.0001 |
| Unclear | -13.1798 | 441.3718 | 0.0000 | Inf | 0.9762 |
| Hypertension | | | | | |
| Yes | Reference | | | | |
| No | -0.5112 | 0.2557 | 0.3633 | 0.9901 | 0.0456 |
| ALT(U/L) | | | | | |
| Male:≥26,Female:>22 | Reference | | | | |
| Male:<26, Female≤22 | -0.7835 | 0.2579 | 0.2755 | 0.7573 | 0.0024 |
| Unclear | -0.8568 | 0.3829 | 0.2004 | 0.8992 | 0.0253 |
| ALB (g/dl) | | | | | |
| ≤4.0 | Reference | | | | |
| >4.0 | -0.9137 | 0.2238 | 0.2586 | 0.6218 | <0.0001 |
| Unclear | -0.8413 | 0.3549 | 0.2151 | 0.8644 | 0.0178 |
| ALP (IU/L) | | | | | |
| ≤129 | Reference | | | | |
| >129 | -0.1884 | 0.2285 | 0.5293 | 1.2963 | 0.4097 |
| Unclear | -0.2792 | 0.3373 | 0.3905 | 1.4650 | 0.4077 |
| LDH（IU/L） | | | | | |
| ≤145 | Reference | | | | |
| >145 | 0.4572 | 0.3513 | 0.7935 | 3.1447 | 0.1931 |
| Unclear | -0.0467 | 0.2989 | 0.5312 | 1.7146 | 0.8758 |
| HbA1c (%) | 0.2037 | 0.1398 | 0.9322 | 1.6123 | 0.1450 |
| LYM (1000 cells/UL) | 0.3180 | 0.1461 | 1.0321 | 1.8301 | 0.0295 |
| NEU (1000 cells/UL) | 0.5031 | 0.5699 | 0.5412 | 5.0537 | 0.3774 |
| MON(1000 cells/UL) | 0.0032 | 0.0645 | 0.8841 | 1.1383 | 0.9603 |
| EOS(1000 cells/UL) | 0.3611 | 0.6173 | 0.4279 | 4.8117 | 0.5586 |
| HGB（g/dl） | 0.0413 | 0.0761 | 0.8977 | 1.2099 | 0.5875 |
| PLT (1000 cells/UL) | -0.0004 | 0.0018 | 0.9960 | 1.0032 | 0.8301 |
| Ferritin (ng/ml) | 0.0054 | 0.0016 | 1.0022 | 1.0085 | 0.0008 |
| HsCRP(mg/L) | | | | | |
| <5 | Reference | | | | |
| ≥5 | 1.2508 | 0.2592 | 2.1018 | 5.8059 | <0.0001 |
| Unclear | -0.0197 | 0.3336 | 0.5098 | 1.8854 | 0.9528 |
| TG (mg/dl) | | | | | |
| ≤150 | Reference | | | | |
| >150 | 0.7399 | 0.2774 | 1.2167 | 3.6095 | 0.0077 |
| Unclear | -0.1053 | 0.3321 | 0.4694 | 1.7258 | 0.7513 |
| HDL(mg/dl) | | | | | |
| ≤50 | Reference | | | | |
| >50 | -0.5444 | 0.2258 | 0.3727 | 0.9032 | 0.0159 |
| Unclear | -0.2827 | 0.3281 | 0.3962 | 1.4339 | 0.3889 |

Supplementary Table 3. The final included covariates

| **Questionnaire information** | | | | |
| --- | --- | --- | --- | --- |
| Gender | Age | Race | Diabetes or Prediabetes | PIR |
| Hypertension |  |  |  |  |
| **Testing information** | | | | |
| ALT | ALB | HbA1c | LYM | MON |
| NEU | HGB | PLT | Ferritin | HsCRP |
| TG | HDL |  |  |  |
